# Impact of socio-demographic and ethnic determinants in guideline-directed medical therapy implementation during heart failure hospitalization

**DOI:** 10.1093/ehjopen/oeaf149

**Published:** 2025-11-04

**Authors:** Renzo Laborante, Agni Delvinioti, Federica Tomassini, Donato Antonio Paglianiti, Gaetano Rizzo, Giuseppe Ciliberti, Attilio Restivo, Jacopo Lenkowicz, Antonio Iaconelli, Stefano Patarnello, Giuseppe Patti, Francesco Canonico, Antonio Gasbarrini, Vincenzo Valentini, Alfredo Cesario, Giovanni Arcuri, Gianluigi Savarese, Filippo Crea, Stefania Boccia, Domenico D’Amario

**Affiliations:** Department of Clinical Science and Education, Södersjukhuset; Karolinska Institutet, Stockholm, Sweden; Department of Cardiovascular and Pulmonary Sciences, Catholic University of the Sacred Heart, Largo A. Gemelli, 00168 Rome, Italy; Gemelli Generator, Fondazione Policlinico Universitario A. Gemelli IRCCS, Largo F. Vito, 00168 Rome, Italy; Gemelli Generator, Fondazione Policlinico Universitario A. Gemelli IRCCS, Largo F. Vito, 00168 Rome, Italy; Department of Cardiovascular and Pulmonary Sciences, Catholic University of the Sacred Heart, Largo A. Gemelli, 00168 Rome, Italy; Department of Translational Medicine, University of Eastern Piedmont, Padiglione A, Largo Bellini, 28100 Novara, Italy; Department of Cardiovascular and Pulmonary Sciences, Catholic University of the Sacred Heart, Largo A. Gemelli, 00168 Rome, Italy; Department of Cardiovascular and Pulmonary Sciences, Catholic University of the Sacred Heart, Largo A. Gemelli, 00168 Rome, Italy; Gemelli Generator, Fondazione Policlinico Universitario A. Gemelli IRCCS, Largo F. Vito, 00168 Rome, Italy; Department of Cardiovascular and Pulmonary Sciences, Fondazione Policlinico Universitario Agostino Gemelli IRCCS, Rome, Italy; Gemelli Generator, Fondazione Policlinico Universitario A. Gemelli IRCCS, Largo F. Vito, 00168 Rome, Italy; Department of Translational Medicine, University of Eastern Piedmont, Padiglione A, Largo Bellini, 28100 Novara, Italy; Thoracic-Cardiovascular Department, Azienda Ospedaliero-Universitaria Maggiore Della Carità, Viale Mazzini, 28100 Novara, Italy; Unit of Internal Medicine, Department of Medical and Surgical Sciences, IRCCS ‘A. Gemelli’ University Polyclinic Foundation, Largo A. Gemelli, 00168 Rome, Italy; Department of Medical and Surgical Sciences, Università Cattolica di Roma, 00168 Rome, Italy; Department of Diagnostic Imaging, Oncological Radiotherapy and Hematology, Catholic University of the Sacred Heart, 00168 Rome, Italy; Gemelli Digital Medicine & Health Srl, Rome, Italy; Gemelli Generator, Fondazione Policlinico Universitario A. Gemelli IRCCS, Largo F. Vito, 00168 Rome, Italy; Department of Clinical Science and Education, Södersjukhuset; Karolinska Institutet, Stockholm, Sweden; Center of Excellence in Cardiovascular Sciences, Ospedale Isola Tiberina, Gemelli Isola, 00100 Roma, Italy; Section of Hygiene, Department of Life Sciences and Public Health, Università Cattolica del Sacro Cuore, 00168Rome, Italy; Department of Women and Child Health and Public Health, Fondazione Policlinico Universitario A. Gemelli IRCCS, 00168 Rome, Italy; Department of Translational Medicine, University of Eastern Piedmont, Padiglione A, Largo Bellini, 28100 Novara, Italy

**Keywords:** Heart failure, Guideline-directed medical therapy

## Abstract

**Aims:**

To assess for the first time the impact of socio-demographic variables on prescription of guideline-directed medical therapy (GDMT) after an episode of heart failure (HF) decompensation in the Italian healthcare system.

**Methods and results:**

Utilizing ‘GENERATOR-HF DataMart’, a cross-sectional analysis was performed. We included patients with HF and reduced ejection fraction discharged between January 2019 and July 2024. The degree of GDMT implementation across the different socio-demographic variables (i.e. patient's age, sex, marital status, nationality, place of residence, and educational level) was evaluated through the modified optimal medical therapy (mOMT) score (i.e. a ratio between the number of pillars actually prescribed and the number of pillars that could be prescribed on the basis of each specific contraindication). A multivariable logistic regression model was also fitted to assess the association between the socio-demographic variables and the prescription of each pillar and loop diuretics. 1730 patients (median age: 72 years; 24% females) were included. The mOMT score was significantly lower in elderly patients, but comparable across other pre-specified socio-demographic categories. In multivariable regression analysis, older age was the only independent socio-demographic predictor of under-prescription both overall and for ACEi/ARB/ARNI (OR0.70; 95% CI 0.55–0.89), beta-blockers (OR0.59; 95% CI 0.41–0.84) and SGLT2i (OR0.66, 95% CI 0.47–0.93), while also associated with a loop diuretics use (OR1.56; 95% CI 1.13–2.17). A higher mOMT score was significantly associated with a reduced incidence of early adverse events (i.e. 30-day all-cause death and urgent re-admissions) (4.1% vs. 8.5%; *P* = 0.001).

**Conclusion:**

Older age was the only independent predictor of under-prescription of GDMT and enhanced use of loop diuretics, whereas no discrepancies were found across the other socio-demographic subgroups.

## Graphical Abstract

**Graphical Abstract:**
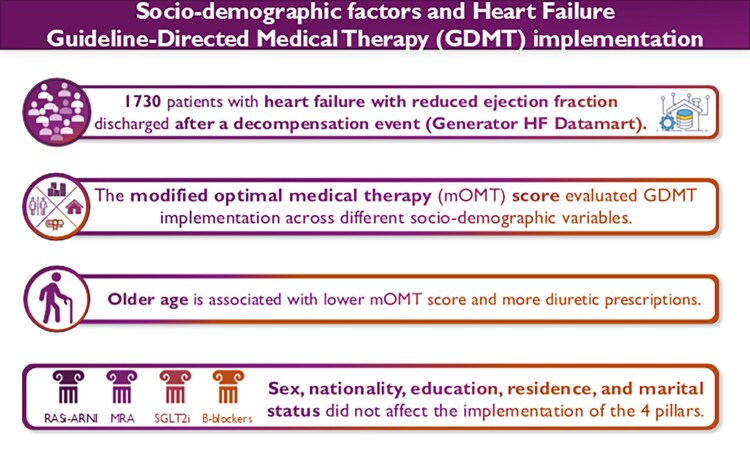

## Introduction

Recent advancements in the treatment of heart failure with reduced ejection fraction (HFrEF) have significantly improved patient outcomes, with both pharmacological and device-based innovations.^1^ The ‘four pillars’ of guideline-directed medical therapy (GDMT) sodium-glucose cotransporter-2 inhibitors (SGLT2i), renin-angiotensin-system inhibitors (RASi) or angiotensin receptor neprilysin inhibitors (ARNI), beta-blockers, and mineralocorticoid receptor antagonists (MRA) have been shown to reduce cardiovascular mortality or hospitalization for heart failure (HF) by 64%.^2^

Despite these proven benefits, the implementation of GDMT in clinical practice remains suboptimal due to various factors, in particular, therapeutic inertia.^3–6^ The impact of social, educational, and ethnic characteristics on GDMT prescription is still debated. While some studies reported treatment disparities across different patient subgroups, others found no significant differences.^7–11^ Many of these studies predate the widespread use of SGLT2 is and did not consider patient contraindications and eventual tolerability issues. Additionally, none of the current studies examined the prescribing practices within the Italian healthcare system, which is based on a universal access model.^12^ Therefore, it is unclear whether disparities exist in GDMT prescriptions at discharge following an acute decompensation episode for HFrEF patients in Italy. Identifying patient subgroups receiving suboptimal care despite similar clinical characteristics could help targeting interventions to reduce therapeutic inertia, offering interpretations on the poor prognoses observed in certain socio-economic or ethnic groups, fostering initiatives able to promote a more inclusive healthcare system.^13,14^

Therefore, our study aimed to evaluate differences in GDMT prescribing practices at discharge, while accounting for contraindications, across socio-demographic subgroups in an Italian tertiary centre during the current era of GDMT.

## Methods

The data that support the findings of this study are available on request from the corresponding author after publication.

### Data extraction, conversion, and validation: gemelli generator HF DataMart framework

To perform this research, the GENERATOR HF DataMart has been used, utilizing an AI-driven process to automatically extract data from diverse sources, enabling the generation of real-world clinical evidence.^5,15^ Briefly, ‘Gemelli Generator HF DataMart’ is an advanced and high-performance ongoing technological infrastructure, aimed at collecting clinical, laboratory, imaging, and on-site contact data of HF patients treated at Fondazione Policlinico A. Gemelli IRCCS starting from 2019, in accordance with the CODE-electronic health record framework.^16,17^ In the present observational study, HF patients were selected based on diagnosis codes listed in Supplementary material online, *Table S1*. Four physicians (R.L., D.A.P., G.C., and A.R.) manually validated the data extracted for the entire cohort of patients to guarantee their accuracy.

### Sample population

We performed a retrospective cross-sectional analysis focusing on consecutive HFrEF patients discharged between January 2019 and July 2024, and identified through the International Classification of Diseases, Ninth Revision (ICD-9) codes 428.*, 402.*, 410.*, 411.*, 413.*, 414.*, 424.*, 425.*, 426.* and 427.*, and at least one echocardiogram showing an ejection fraction (EF) of less than 40% (see Supplementary material online, *Table S1*). In the case of multiple records, we selected the first one with HF as the primary diagnosis. Patients who died during hospitalization were excluded. The reliability of ICD-9 code assignment was ensured by adherence to Joint Commission International standards. Cohort selection and subsequent analysis were approved by the local ethics committee.

### Variables

From the Gemelli Generator HF Data Mart, 43 variables were selected, including administrative and structured clinical data. Contraindications to RASi, ARNi, β-blockers, MRA, and SGLT2i were considered according to the 2021 ESC Guidelines on HF (see Supplementary material online, *Table S2*). A detailed description of the variables used to define contraindications was provided in the Supplementary material online, *Table S3*. Sociodemographic variables, i.e. age (i.e. > or ≤ 75 years), sex (male vs. female), civil status (married or living with someone vs. living alone), nationality (Italian vs. non-Italian), place of residence (provincial capital vs. regional capital vs. others), level of education (none vs. primary vs. secondary vs. higher) were automatically extracted from the Electronic Health Record (EHR). In case of missing data for socio-demographic variables, patients were contacted by telephone by 5 investigators (DAP, GC, AR, GR, FT). To assess the degree of GDMT implementation at discharge, we used the ratio calculated as a percentage [i.e. modified optimal medical therapy (mOMT) score] with the number of pillars actually prescribed as numerator and the number of pillars that could have prescribed while also considering specific contraindications as denominator. Given the change in availability in lifesaving GDMT for HFrEF over time, patients were stratified based on their discharge date into 2 groups: before and after June 2022. Prior to June 2022, three GDMTs were available for prescription, i.e. beta-blockers, ACEi/ARB/ARNI, and MRA. SGLT2i were therefore not considered in the score calculation before this date. From June 2022 onward, SGLT2i were also considered given their approval for reimbursement in Italy for HF. Only drugs with Class I recommendation according to European HF guidelines were selected for the score.^1^ Missing data were handled using iterative multiple imputation by chained equations. The imputation model included socio-demographic characteristics, comorbidities, clinical and laboratory variables, excluding those with a high proportion of missing values (i.e. > 25%) (marked with *in *Table 1*). The rate of the composite outcome of 30-day all-cause death and 30-day urgent readmissions, as well as of the individual components, was assessed according to mOMT score levels (≤ median vs. > median) and compared using the Chi-square test.

**Table 1:** Baseline characteristics of the overall population

Variables	Total patients (*n* = 1730)	Missing values
**Demographics/organizational/socioeconomic**		
Age (years), median (IQR)^a^	72.0 (62.0,80.0)	0 (0.0%)
Male sex	1315 (76.0%)	0 (0.0%)
Married/Living with someone^a^	936 (54.1%)	415 (24.0%)
Nationality^a^		0 (0.0%)
Foreign	93 (5.4%)	
Italian	1637 (94.6%)	
Permanent address^a^		14 (0.8%)
In a region capital	932 (53.9%)	
In a province capital	65 (3.8%)	
Other	719 (41.6%)	
Education^a^		404 (23.4%)
No education	30 (1.7%)	
Primary	208 (12.0%)	
Secondary	857 (49.5%)	
Higher	231 (13.4%)	
Discharged		0 (0.0%)
Before June 2022	1033 (59.7%)	
After June 2022	697 (40.3%)	
**Clinical**		
NYHA		1392 (80.5%)
I	15 (0.9%)	
II	80 (4.6%)	
III	192 (11.1%)	
IV	51 (2.9%)	
Heart rate (bpm), median (IQR)	74.0 (68.0,80.0)	451 (26.1%)
AV block		0 (0.0%)
I	98 (5.7%)	
II	39 (2.3%)	
III	49 (2.8%)	
Systolic blood pressure (mmHg), median (IQR)	114.0 (105.0, 125.0)	331 (19.1%)
BMI (kg/m2), median (IQR)	25.7 (24.0,28.4)	307 (17.7%)
**Laboratory values**		
Hemoglobin(g/dL), median (IQR)^a^	12.2 (10.0,14.1)	7 (0.4%)
NT-proBNP (pg/mL), median (IQR)^a^	3215.0 (1320.0, 7310.5)	331 (19.1%)
eGFR (mL/min/1.73 m2), median (IQR)^a^	70.0 (50.0,89.0)	7 (0.4%)
Potassium (mEq/L), median (IQR)	4.0 (4.0,4.2)	9 (0.5%)
**History and comorbidities**		
Diabetes^a^	847 (49.0%)	0 (0.0%)
Pulmonary disease	457 (26.4%)	0 (0.0%)
Malignant disease^a^	384 (22.2%)	0 (0.0%)
Hypertension	1206 (69.7%)	0 (0.0%)
Hepatic disease	21 (1.2%)	0 (0.0%)
**Treatment**		
β-blockers	1595 (92.2%)	0 (0.0%)
ACEi	277 (16.0%)	0 (0.0%)
ARB	190 (11.0%)	0 (0.0%)
ARNi	823 (47.6%)	0 (0.0%)
MRA	503 (29.1%)	0 (0.0%)
SGLT2i	373 (21.6%)	0 (0.0%)
Diuretics	1524 (88.1%)	0 (0.0%)
Digoxin	75 (4.3%)	0 (0.0%)
Statin	1013 (58.6%)	0 (0.0%)
Acetylsalicylic acid (ASA)	696 (40.2%)	0 (0.0%)
Furosemide	1341 (77.5%)	0 (0.0%)
**Outcomes**		
mOMT > 67%	640 (37.0%)	0 (0.0%)
Urgent readmission within 30 days	74 (4.3%)	0 (0.0%)
Death within 30 days	49 (2.8%)	0 (0.0%)
Urgent readmission or death within 30 days	119 (6.9%)	0 (0.0%)

Abbreviations: ACEi, angiotensin-converting enzyme inhibitors; ARB, angiotensin receptor blockers; ARNi, angiotensin receptor–neprilysin inhibitor; MRA, mineralocorticoid receptor antagonists; SGLT2i, Sodium-glucose co-transporter 2 inhibitors; AV, atrioventricular; NYHA, New York Heart Association.

^a^Included in multiple imputation model and adjusted for in logistic regression models.

### Statistical analysis

Continuous variables were reported as median and interquartile range (IQR), and categorical variables were reported as counts and proportions (percentages). Baseline characteristics were compared across subgroups using the Kruskal-Wallis test for continuous variables and Pearson χ^2^ test for categorical variables. These non-parametric tests were used given the non-normal distributions of the variables, assessed through the Kolmogorov-Smirnov test. Statistical analysis was performed using Python version 3.11.5 with main packages being: *pandas* for data processing, *scipy* for statistical tests, *statsmodels* for modelling, *matplotlib*, *scikit-learn* for data imputation, and *zepid* for reporting. We used SAS for extracting, transforming, loading data extraction, and text mining pipelines. A *P* < 0.05 (two-tailed) was considered statistically significant.

### Secondary analyses

The independent association between the sociodemographic characteristics of interest and the prescription of each pillar and loop diuretics at discharge was assessed by a multivariable logistic regression model, including the variables marked with ^a^ in *Table 1*. For SGLT2i, predictors of prescription were assessed after June 2022 (when both empagliflozin and dapagliflozin became reimbursable in Italy for HF), while for the remaining pillars and loop diuretics the entire cohort was considered.

## Results

**Figure 1:**
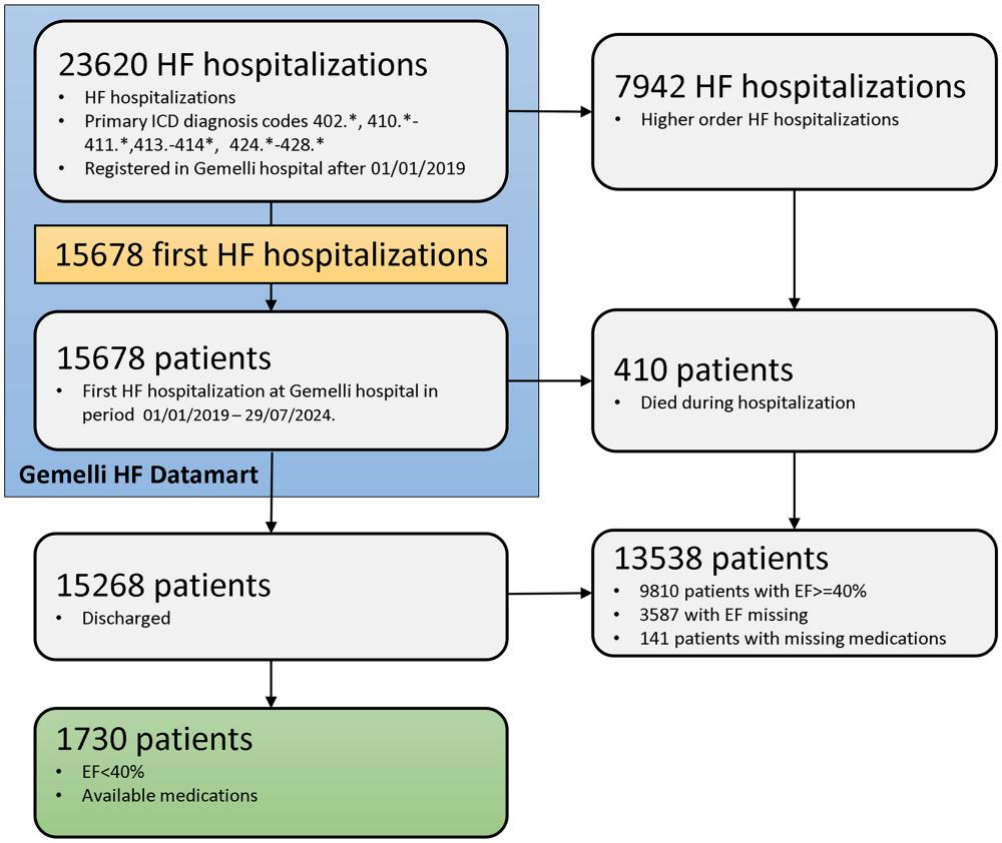
Flowchart of cohort selection. Abbreviations: EF, ejection fraction; HF, heart failure; ICD, International Classification of Diseases.

From January 2019 to July 2024, the Gemelli Generator HF Data Mart included 23 620 HF patients discharged after a HF decompensation event. Of these, 1730 patients were considered for the present analysis after applying the selection criteria of this study (*Figure 1*). The baseline characteristics of the entire population were shown in *Table 1*. The median age of the overall population was 72 years (IQR, 62–80 years), 76% of patients were males, and 95% of them were Italian. Missing values for baseline characteristics were reported in *Table 1*, whereas the prevalence of contraindications/cautions and related missing values were reported in Supplementary material online, *Table S4* and Supplementary material online, *Table S5*, respectively.

### Prescription and prescriptibility of the ‘four pillars’ at discharge

**Figure 2:**
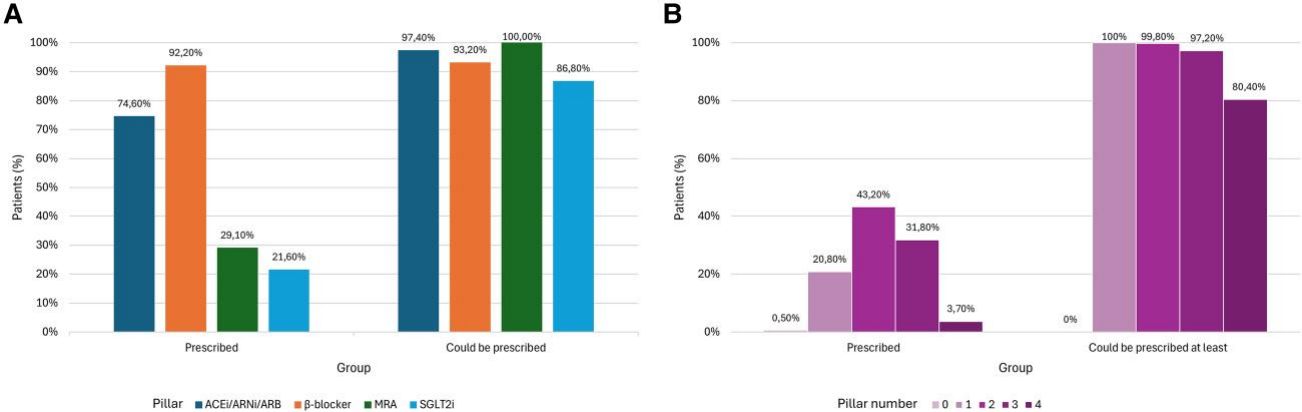
Type (*A*) and number (*B*) of pillars prescribed and that could be prescribed based on contraindications. Abbreviations: ACEi, angiotensin-converting enzyme inhibitor; ARB, angiotensin receptor blockers; ARNI, angiotensin receptor neprilysin inhibitor; MRA, mineralocorticoid receptor antagonist; SGLT2i, sodium-glucose cotransporter-2 inhibitor.

At discharge, 92% of patients received a beta-blocker, 75% were prescribed an ACEi/ARB/ARNI, 29% an MRA, and 22% a SGLT2i. However, 97%, 93%, 100% and 87% of HFrEF patients could have received at discharge ACEi/ARB/ARNI, beta-blocker, MRA, SGLT2i, respectively, based on their respective contraindications to treatments (*Figure 2A*). Moreover, only 36% of the study patients received three or four pillars, while 97% of them could have received at least three pillars at discharge (*Figure 2B*).

### Modified optimal medical therapy score

**Figure 3:**
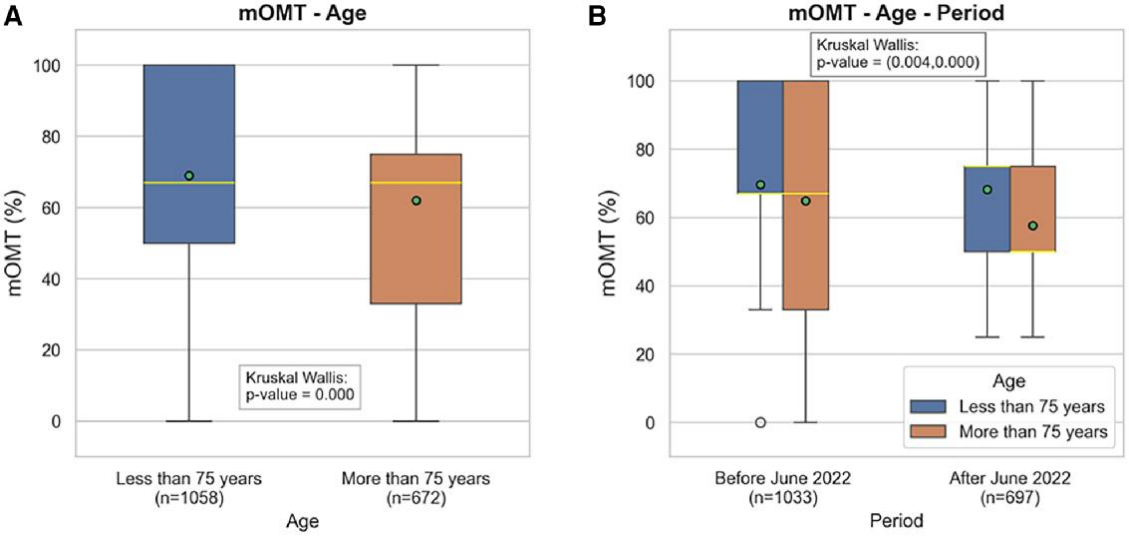
Distributions of the mOMT score according to age, overall (*A*) and in the two-time sub-groups (*B*). Abbreviation: mOMT, modified optimal medical therapy.

**Figure 4:**
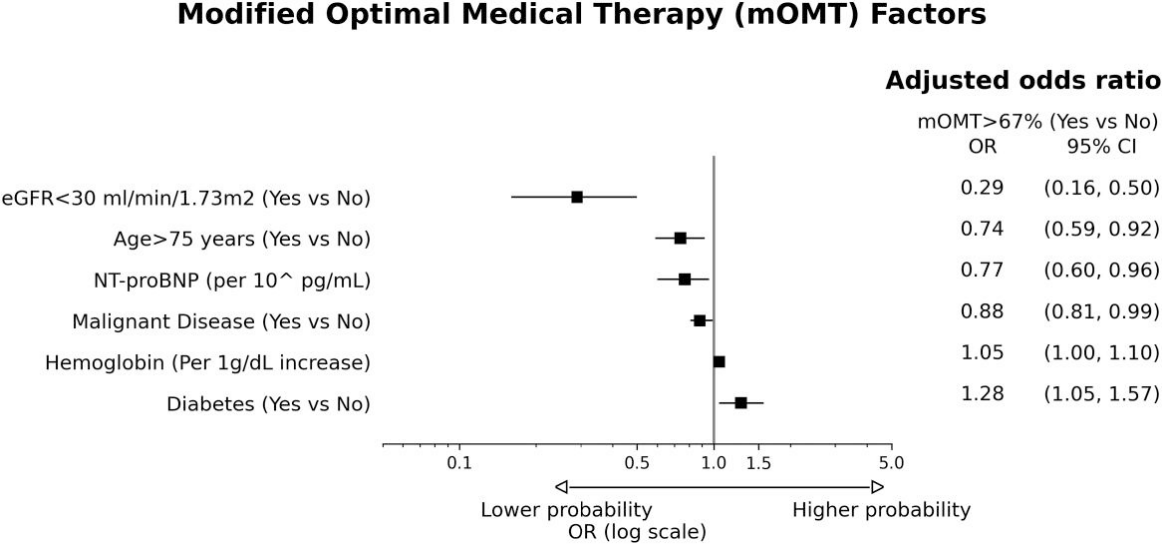
Factors independently associated with a lower mOMT score. Abbreviation: CI, confidence interval; eGFR, estimated glomerular filtration rate; log, logarithmic; NT-proBNP, N-terminal pro–B-type natriuretic peptide; mOMT, modified optimal medical therapy; OR, odd ratio.

Overall, the median mOMT score was 67% (IQR: 50–75%) and was comparable in the two-time subgroups [median 2019–2022: 67% (IQR 50–100%), median 2022–2024: 67% (IQR: 50–75%), *P* = 0.208] and across sexes, place of residence (i.e. provincial capital vs. regional capital vs. others), civil status, education level, and nationality categories (see Supplementary material online, *Figure S1*). Conversely, older patients (i.e. > 75 years) scored significantly lower both in the overall cohort (*Figure 3A*) and in the two-time subgroups (*Figure 3B*). For the multivariable regression model, the dependent variable was defined as dichotomous, with a mOMT score > or ≤ 67%, corresponding to the median value in the entire cohort. Among the pre-specified socio-demographic variables, older age (i.e. > 75 years) was found to be the only independent predictor of under-prescription of GDMT (adjusted OR: 0.74; 95% CI: 0.59–0.92; *P* < 0.001) (*Figure 4*).

### Socio-demographic predictors of prescription for each pillar and loop diuretics

**Figure 5:**
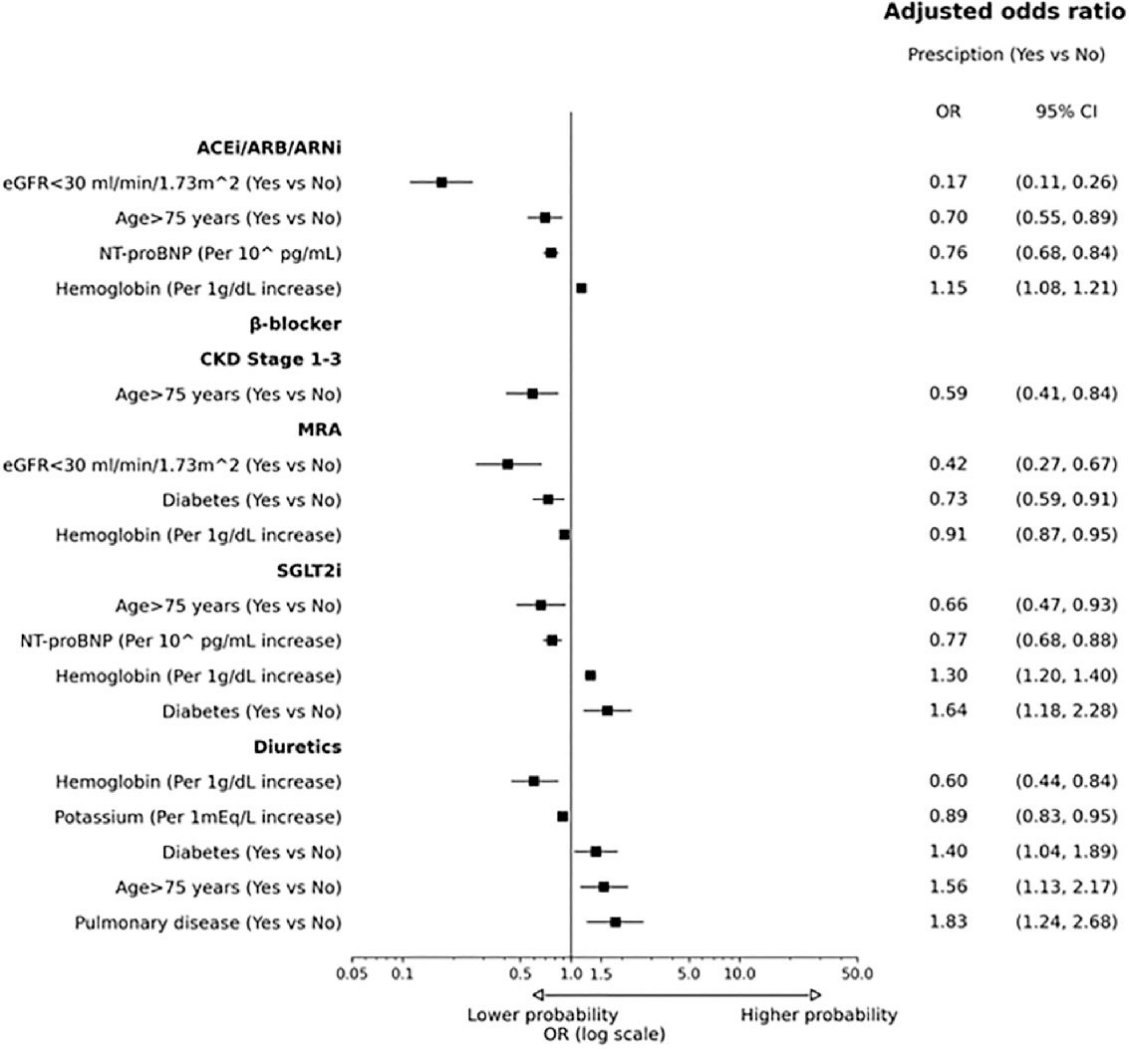
Factors independently associated with prescription of each pillar and loop diuretics. Abbreviation: ACEi, angiotensin-converting enzyme inhibitor; ARB, angiotensin receptor blockers; ARNI, angiotensin receptor neprilysin inhibitor; CI, confidence interval; CKD, chronic kidney disease; eGFR, estimated glomerular filtration rate; NT-proBNP, N-terminal pro-B-type natriuretic peptide; log, logarithmic; mOMT, modified optimal medical therapy; MRA, mineralocorticoid receptor antagonist; OR, odd ratio; SGLT2i, sodium-glucose cotransporter-2 inhibitor.

Among the pre-specified socio-demographic variables, older age (i.e. > 75 years) was the only independent predictor of non-prescription for beta-blockers (OR: 0.59; 95% CI: 0.41–0.84; *P* < 0.001), ACEi/ARB/ARNI (OR: 0.70; 95% CI: 0.55–0.89; *P* < 0.001), and SGLT2i (OR: 0.66; 95% CI: 0.47–0.93; *P* = 0.02) in eligible patients (*Figure 5*, Supplementary material online, *Table S6*–S9). Regarding MRAs, none of the socio-demographic variables independently predicted under-prescription in eligible patients (*Figure 5*) (see Supplementary material online, *Table S8*). Regarding loop diuretics, older age (i.e. > 75 years) was the only variable positively associated with their prescription after multivariable adjustments (OR 1.56; 95% CI; 1.13, 2.17; *P* < 0.01) (*Figure 5*) (see Supplementary material online, *Table S10*). Calibration plots for the outcomes mOMT >67% and ACEi/ARB/ARNi were provided, showing that model predictions were well calibrated with respect to the observed proportion of positive cases (see Supplementary material online, *Figure S2*).

### Clinical outcomes

At 30 days, 6.9% of enrolled patients were re-hospitalized for urgent causes or died. Patients in the higher-score group (mOMT score > median) were associated with a lower risk of early adverse events (4.1% vs. 8.5%; *P* = 0.001), primarily driven by a lower risk of all-cause death (0.9% vs. 3.9%; *P* < 0.001) (*Table 2*).

**Table 2:** Early adverse clinical events

Outcome	Patients (*n* = 1730)		Chi-square test
	mOMT score ≤67% (*n* = 1090)	mOMT score >67% (*n* = 640)	*P*-value
Urgent all cause re-admission within 30 days	52 (4.8%)	22 (3.4%)	0.230
Death within 30 days	43 (3.9%)	6 (0.9%)	0.000
Urgent all cause readmission or death within 30 days	93 (8.5%)	26 (4.1%)	0.001

Abbreviations: mOMT, modified optimal medical therapy.

## Discussion

The main findings of the current work, including 1730 patients with a first diagnosis of HFrEF at discharge after an episode of decompensation, were the following: 1) the vast majority of patients with HFrEF could be prescribed three or four pillars at discharge, but only a modest percentage received them (i.e. 97% vs. 36%); 2) older age (i.e. > 75 years) was the only sociodemographic predictor of overall under-prescription of the four pillars, defined according to the mOMT score; 3) older age (i.e. > 75 years) remained the only sociodemographic independent predictor of under-prescription for beta-blockers, ACEi/ARB/ARNI, and SGLT2i, but, 4) was the only socio-demographic factor positively associated with loop diuretic prescription at discharge; 5) a higher mOMT score was significantly associated with a reduced incidence of early adverse events. Overall, our study highlighted that in a universal healthcare system, in-hospital access to care for HF patients was not influenced by socio-demographic factors (i.e. sex, marital status, educational level, nationality, place of residence), suggesting equitable healthcare access. However, further studies are needed to assess whether this equality persists during follow-up, nullifying difference in the incidence of clinical adverse events.

### Age

Numerous European and non-European registries have consistently demonstrated a significant underuse of GDMT in older patients with HFrEF, both in the acute hospitalization setting and in outpatient care.^18,19^ However, the majority of existing evidence primarily pertains to the underuse of beta-blockers, ACEi/ARBs, ARNIs, and MRAs, with relatively limited data on the real-world utilization of SGLT2i in this cohort, given their recent clinical adoption.^20^ To date, our study is the first observational analysis specifically investigating the impact of age on the real-world prescription of GDMT at discharge following an acute decompensation event in HFrEF patients in the Italian healthcare system. Notably, our findings indicate that, in elderly patients without absolute contraindications, the under-prescription predominantly affects beta-blockers, ACEi/ARB/ARNI, and SGLT2i, while the prescription of MRAs remains consistently low (≈ 30%) across all age groups, likely reflecting poor prescribing patterns even in younger patients. Since individuals with absolute contraindications were excluded, it appears that therapeutic inertia is the predominant factor contributing to this under-prescription,^3^ particularly affecting MRAs and SGLT2i. While the inertia observed with the latter may be attributable to their recent introduction, the inertia regarding MRAs is alarming and necessitates urgent and targeted interventions to assist clinicians in overcoming this barrier.^15^

The underrepresentation of elderly patients in large randomized clinical trials may contribute to clinicians’ hesitancy in prescribing these therapies, particularly in the post-hospitalization setting, due to the limited evidence supporting their use.^21^ Additionally, the complex multi-morbidities pattern which characterizes often the older population, coupled with concerns regarding polypharmacy, may further hinder the comprehensive initiation of GDMT in this subgroup.^22^ Nevertheless, a recent post-hoc analysis of the STRONG-HF trial demonstrated that up-titration of beta-blockers, ACEi/ARB/ARNI, and MRAs post-hospitalization for HF was equally effective and safe in patients over 65 years of age as in younger patients.^23^ Similarly, a recent meta-analysis on the efficacy and safety of SGLT2i in acute HF confirmed their safety, with no significant interaction related to patient age.^24^

In our analysis, elderly patients were more frequently prescribed loop diuretics, even after adjusting for potential confounders such as comorbidities and *n*-terminal pro–B-type natriuretic peptide levels. This observation aligns with findings from other studies across different countries and time periods.^19,25–27^ The preference for loop diuretics in older adults may stem from a greater focus on symptom relief rather than long-term prognostic improvement. However, their indiscriminate and widespread use can lead to adverse outcomes, including sympathetic hyperactivation, hypotension, renal dysfunction, and electrolyte imbalances.^6,28^ Given the debated and generally unfavourable prognostic impact of loop diuretics on long-term outcomes, their use should be approached with caution, especially when weighed against the well-established prognostic benefits of GDMT.^6,29,30^ Given the rising average age of HF patients, particularly in Western countries, there is an urgent need for interventions to increase the uptake of GDMT, with a specific focus on elderly patients, within the Italian healthcare system.^31^

### Educational level

Among the predictors of four pillars prescription, the impact of educational level has been poorly investigated.^7,20,21^ In the CHAMP-HF registry, including 3518 outpatients in the United States patients with chronic HFrEF, the prescription of ACE/ARB/ARNi, beta-blockers and MRAs and their dosage was not independently associated with the educational level.^7^ Conversely, in a study including 336 Asian patients hospitalized for HFrEF, a high education attainment was significantly associated with a 22% higher likelihood of receiving GDMT (i.e. beta-blockers, ACEi/ARB/ARNI, and MRA) at discharge.^32^ Similarly, an analysis from the Swedish HF Registry, encompassing 8192 patients with acute and chronic HFrEF from 2020 to 2022, showed a positive association between higher levels of education and SGLT2i prescription.^20^ In our analysis, the education level was not associated with the overall GDMT prescription, defined on the basis of the mOMT score, nor with the prescription of the individual pillars, including the latest SGLT2i. To our knowledge, our study represents the first study to assess the impact of patient education level on GDMT adoption at discharge in the Italian healthcare system. Our finding seems to suggest that a national healthcare system based on the Beveridge model, with full reimbursement of disease-modifying drugs for every citizen, nullifies the differences in GDMT prescription between patients from different levels of education. Nonetheless, additional analyses from other Italian centres are needed to validate and generalize this initial finding.

### Sex

Females represent a minority in cardiovascular research, including in the HF-related field, with under-representation (i.e. < 25% of total enrolled patients) in randomized controlled trials.^33,34^ Data on the underuse of GDMT in females with HFrEF in real-world studies are inconsistent and vary across the different pillars. In two European registries, after extensive adjustment for potential confounders, females received similar prescriptions for ACEi/ARB/ARNI, MRA, and beta-blockers, but were less likely to receive SGLT2i as compared to males.^20,35,36^ In contrast, in three North American registries, females exhibited less use of GDMT in terms of beta-blockers, ACEi/ARB/ARNI and MRA.^37–39^ Notably, among patients with Medicare insurance, the utilization rates of optimal GDMT were comparable between sexes, in contrast to those with private insurance.^38^ In accordance with other European registries, our study showed similar implementation of GDMT across sexes, suggesting that a public health system may have contributed to closing the sex gap in the access to treatment for HF patients.

### Place of residence

Some reported lower prescription of GDMT in HF patients living in rural centres as compared with those from urban centres.^9,40^ This may be attributable to more limited access to healthcare services and less intensive follow-up, thereby hindering the implementation of GDMT post-discharge. In contrast, our current study found that the place of residence did not appear to influence the prescription of GDMT at discharge, although differences in implementation and adherence after discharge cannot be fully ruled out.

### Civil status

HF is a chronic condition, associated with a significant burden on patients and their families because it requires multiple daily medications, exercise, dietary modifications, and frequent clinical appointments.^41^ Some studies reported a worse prognosis for patients with cardiovascular disease who live alone than for those who are married.^41,42^ Whether marital status influences clinician prescription appropriateness has been little investigated in the contemporary era of GDMT. Our study showed, for the first time in the Italian healthcare system, that the prescription of GDMT was not affected by the patient's marital status. Living alone was not perceived *per se* as a barrier to implementing GDMT at discharge.

### Nationality

Ethnic minorities with HF are more likely to experience a poorer prognosis than Caucasian patients in high-income countries.^14,43^ The reason for this difference is not well understood. Some studies showed a lower prescription of GDMT in ethnic minorities, especially for newer drugs (i.e. SGLT2i).^44^ Nevertheless, the appropriateness of prescribing GDMT according to birthplace in the Italian healthcare system has never been evaluated. In our study, non-Italian patients receive comparable management regarding overall GDMT prescription, as shown by a similar mOMT score, as well as in the individual components. Overall, our study seems to suggest the absence of nationality-related inequalities in access to HF medications.

## Strength and limitations

The main strength of this study is that it represents the first in Italy to assess the impact of socio-demographic factors on GDMT uptake at discharge after HF hospitalization in a large, real-world cohort of HFrEF patients. Nonetheless, several limitations should be acknowledged. First, the study was conducted at a single tertiary centre, which may introduce selection bias, limiting generalizability to the broader Italian healthcare system. Nonetheless, the baseline characteristics of the enrolled patients (age, sex, major comorbidities) were comparable to those reported in another contemporary multicenter Italian registry, which may support the generalizability of our findings.^45^ The absence of prior HF hospitalizations in other centres cannot be fully confirmed, as we did not have access to data from institutions outside our own. Second, we did not assess the dosage of GDMT, which is prognostically relevant for some medications (e.g. beta-blockers, ACEi/ARB/ARNI).^46^ Third, due to the observational nature of the study, the presence of residual confounding (i.e. frailty, potassium levels) cannot be entirely ruled out, despite adjustments for numerous clinical and laboratory variables. Since longitudinal data on the prescription trajectories of the GDMT were not available, it is not possible to determine whether under-prescription in older patients is partly attributable to de-prescription driven by frailty and high multimorbidity, which may limit the feasibility of a polypharmacological strategy.

## Conclusions

Our study is the first in Italy to demonstrate the absence of disparities in GDMT prescription based on sex, place of residence, educational level, marital status, and nationality. However, GDMT remains under-prescribed in older adults, with loop diuretics being widely used as an alternative. Targeted strategies to enhance GDMT utilization in the elderly are urgently needed.

## Supplementary Material

oeaf149_Supplementary_Data

## Data Availability

The data underlying this article will be shared on reasonable request to the corresponding author.
