# Supplementary material for: Impact of socio-demographic and ethnic determinants in guideline-directed medical therapy implementation during heart failure hospitalization
Source: Eur Heart J Open. 2025 Nov 4;5(6):oeaf149. doi: 10.1093/ehjopen/oeaf149 (PMC12686985; doi:10.1093/ehjopen/oeaf149)
Supplement: oeaf149_Supplementary_Data [file oeaf149_supplementary_data.docx]

**SUPPLEMENTAL MATERIAL**

**Supplemental Table 1.** ICD-9 code used.

| **ICD-9 code** | **Disease** | **Total patients (n=1730)** |
| --- | --- | --- |
| **428.*** | Heart failure | 889 (51.4%) |
| **402.*** | Hypertensive heart disease | 11 (0.6%) |
| **410.*** | Acute myocardial infarction | 277 (16.0%) |
| **411.*** | Other acute and subacute forms of ischemic heart disease | 103 (6.0%) |
| **413.*** | Angina pectoris | 26 (1.5%) |
| **414.*** | Other forms of chronic ischemic heart disease | 197 (11.4%) |
| **424.*** | Mitral valve disorders | 123 (7.1%) |
| **425.*** | Cardiomyopathy | 19 (1.1%) |
| **426.*** | Conduction disorders | 5 (0.3%) |
| **427.*** | Cardiac dysrhythmias | 80 (4.6%) |

**Supplemental Table 2.**

Contraindications of recommended medications according to the European Society of Cardiology Guidelines on Heart Failure 2021.

| **ACEi/ARNi/ARB** | |
| --- | --- |
| **Contraindications** | |
| History of angioedema |  |
| Bilateral renal artery stenosis |  |
| Pregnancy | Female gender and age less than 50 years |
| ACEi/ARB/ARNi adverse reaction | Known allergic reaction/other adverse reaction to ACEi/ARB/ARNi |
| **β-blocker** | |
| **Contraindications** | |
| AV block II/III without permanent pacemaker | Second or third degree of AV block (in the absence of a permanent pacemaker) |
| Critical limb ischaemia |  |
| Asthma |  |
| β-blocker adverse reaction | Known allergic reaction/other adverse reaction to β-blocker |
| **MRA** | |
| **Contraindications** | |
| MRA adverse reaction | Known allergic reaction/other adverse reaction to MRA |
| **SGLT2i** | |
| **Contraindications** | |
| SGLT2i adverse condition | Known allergic reaction/other adverse reaction to SGLT2i |
| Pregnancy | Female gender and age less than 50 years |
| Signiﬁcant renal dysfunction | eGFR<20 ml/min/1.73m2 |
| Hypotension | Systolic blood pressure<95 mmHg or symptomatic hypotension |

**Abbreviations.** ACEi, angiotensin-converting enzyme inhibitors; ARB, angiotensin receptor blockers; ARNi, angiotensin receptor–neprilysin inhibitor; MRA, mineralocorticoid receptor antagonists; SGLT2i, Sodium-glucose co-transporter 2 inhibitors; AV, atrioventricular; NYHA, New York Heart Association

**Supplemental Table 3.** Variables definition.

| Variable | Definition |
| --- | --- |
| eGFR | Estimated glomerular filtration rate calculated based on CKD-EPI 2021 |
| Pregnancy | Female gender and age less than 50 years |
| ACEi/ARB/ARNi adverse reaction | Known allergic reaction/other adverse reaction to ACEi/ARB/ARNi |
| Signiﬁcant renal dysfunction [ACEi/ARB/ARNi and β-blocker ] | eGFR<30 ml/min/1.73m2 or Creatinine>2.5 mg/dL |
| AV block II/III without permanent pacemaker | Second or third degree of AV block (in the absence of a permanent pacemaker) |
| β-blocker adverse reaction | Known allergic reaction/other adverse reaction to β-blocker |
| MRA adverse reaction | Known allergic reaction/other adverse reaction to MRA |
| SGLT2i adverse condition | Known allergic reaction/other adverse reaction to SGLT2i |
| Signiﬁcant renal dysfunction [SGLT2i] | eGFR<20 ml/min/1.73m2 |
| Hypotension [SGLT2i] | Systolic blood pressure<95 mmHg or symptomatic hypotension |
| All included variables reported at the date of discharge. ACEi, angiotensin-converting enzyme inhibitors; ARB, angiotensin receptor blockers; ARNi, angiotensin receptor–neprilysin inhibitor; MRA, mineralocorticoid receptor antagonists; NYHA, New York Heart Association; NT-proBNP, N-terminal pro hormone brain natriuretic peptide; SGLT2i, Sodium-glucose co-transporter 2 inhibitors; AV, atrioventricular | |
|  |  |

**Supplemental Table 4.** Prevalence of contraindications according to drug type.

| Pillar | Total patients (n = 1730) | | Prescribed | Not prescribed | Missing rate |
| --- | --- | --- | --- | --- | --- |
| ACEi/ARB/ARNi | |  | 1290 (74.6%) | 440 (25.4%) | - |
| History of angioedema | | 9 (0.5%) | 7 (0.5%) | 2 (0.5%) | Not estimable |
| Bilateral renal artery stenosis | | 5 (0.3%) | 3 (0.2%) | 2 (0.5%) | Not estimable |
| Pregnancy | | 24 (1.4%) | 23 (1.8%) | 1 (0.2%) | 0 (0.0%) |
| ACEi/ARB/ARNi adverse reaction | | 7 (0.4%) | 6 (0.5%) | 1 (0.2%) | Not estimable |
| β-blocker | |  | 1595 (92.2%) | 135 (7.8%) | - |
| AV block II/III without permanent pacemaker | | 11 (0.6%) | 11 (0.7%) | 0 (0.0%) | Not estimable |
| Critical limb ischaemia | | 59 (3.4%) | 51 (3.2%) | 8 (5.9%) | Not estimable |
| Asthma | | 43 (2.5%) | 41 (2.6%) | 2 (1.5%) |  |
| β-blocker adverse reaction | | 5 (0.3%) | 4 (0.3%) | 1 (0.7%) | Not estimable |
| MRA | |  | 503 (29.1%) | 1227 (70.9%) | - |
| MRA adverse reaction | | 0 (0.0%) | 0 (0.0%) | 0 (0.0%) | Not estimable |
| SGLT2i | |  | 373 (21.6%) | 1357 (78.4%) | - |
| SGLT2i adverse condition | | 1 (0.1%) | 0 (0.0%) | 1 (0.1%) | Not estimable |
| Pregnancy | | 24 (1.4%) | 9 (2.4%) | 15 (1.1%) | 0 (0.0%) |
| Signiﬁcant renal dysfunction | | 82 (4.7%) | 4 (1.1%) | 78 (5.7%) | 7 (0.4%) |
| Hypotension | | 119 (6.9%) | 40 (10.7%) | 79 (5.8%) | 331 (19.1%) |
| ACEi, angiotensin-converting enzyme inhibitors; ARB, angiotensin receptor blockers; ARNi, angiotensin receptor–neprilysin inhibitor; MRA, mineralocorticoid receptor antagonists; SGLT2i, Sodium-glucose co-transporter 2 inhibitors; AV, atrioventricular | | | | | |

**Supplemental Table 5.** Prevalence of contraindications per period.

| Pillar | Before June 2022 (n = 1033) | | After June 2022  (n=697) | p-value | |
| --- | --- | --- | --- | --- | --- |
| ACEi/ARB/ARNi | | 725 (70.2%) | 565 (81.1%) |  | |
| History of angioedema | | 6 (0.6%) | 3 (0.4%) | 0.747 | |
| Bilateral renal artery stenosis | | 2 (0.2%) | 3 (0.4%) | 0.398 | |
| Pregnancy | | 13 (1.3%) | 11 (1.6%) | 0.676 | |
| ACEi/ARB/ARNi adverse reaction | | 3 (0.3%) | 4 (0.6%) | 0.449 | |
| β-blocker | | 952 (92.2%) | 643 (92.3%) |  | |
| AV block II/III without permanent pacemaker | | 8 (0.8%) | 3 (0.4%) | 0.541 | |
| Critical limb ischaemia | | 40 (3.9%) | 19 (2.7%) | 0.225 | |
| Asthma | | 30 (2.9%) | 13 (1.9%) | 0.208 | |
| β-blocker adverse reaction | | 4 (0.4%) | 1 (0.1%) | 0.654 | |
| MRA | | 357 (34.6%) | 146 (20.9%) |  | |
| MRA adverse reaction | | 0 (0.0%) | 0 (0.0%) | - | |
| SGLT2i | | 51 (4.9%) | 322 (46.2%) |  | |
| SGLT2i adverse condition | | 0 (0.0%) | 1 (0.1%) | 0.403 | |
| Pregnancy | | 13 (1.3%) | 11 (1.6%) | 0.676 | |
| Signiﬁcant renal dysfunction | | 54 (5.2%) | 28 (4.0%) | 0.299 | |
| Hypotension | | 43 (4.2%) | 76 (10.9%) | **0.000** | |
| ACEi, angiotensin-converting enzyme inhibitors; ARB, angiotensin receptor blockers; ARNi, angiotensin receptor–neprilysin inhibitor; MRA, mineralocorticoid receptor antagonists; SGLT2i, Sodium-glucose co-transporter 2 inhibitors; AV, atrioventricular | | | | |  |

**Supplemental Table 6.** β-blocker prescription for patients without contraindications in the overall cohort.

| Variables | Total patients (n = 1612) | Patients with  β-blocker | p-value |
| --- | --- | --- | --- |
| Demographics/organizational/social |  |  |  |
| Age |  |  | **0.010** |
| > 75 years | 638 | 575 (90.1%) |  |
| ≤ 75 years | 974 | 913 (93.7%) |  |
| Sex |  |  | 0.527 |
| Male | 1227 | 1136 (92.6%) |  |
| Female | 385 | 352 (91.4%) |  |
| Civil staus |  |  |  |
| Married/Living with someone | 873 | 804 (92.1%) | 0.497 |
| Not married/Living alone | 350 | 327 (93.4%) |  |
| Nationality |  |  | 0.324 |
| Foreign | 90 | 86 (95.6%) |  |
| Italian | 1522 | 1402 (92.1%) |  |
| Permanent address |  |  | 0.075 |
| In a region capital | 873 | 803 (92.0%) |  |
| In a province capital | 60 | 60 (100.0%) |  |
| Other | 666 | 614 (92.2%) |  |
| Education |  |  | 0.555 |
| No education | 29 | 28 (96.6%) |  |
| Primary | 189 | 171 (90.5%) |  |
| Secondary | 799 | 738 (92.4%) |  |
| Higher | 216 | 202 (93.5%) |  |

**Supplemental Table 7.** ACEi/ARB/ARNI prescription for patients without contraindications in the overall cohort.

| Variables | Total patients (n = 1685) | Patients with ACEi/ARB/ARNI | p-value |
| --- | --- | --- | --- |
| Demographics/organizational/social |  |  |  |
| Age |  |  | **0.000** |
| > 75 years | 660 | 443 (67.1%) |  |
| ≤ 75 years | 1025 | 808 (78.8%) |  |
| Sex |  |  | 0.779 |
| Male | 1303 | 970 (74.4%) |  |
| Female | 382 | 281 (73.6%) |  |
| Civil staus |  |  |  |
| Married/Living with someone | 916 | 663 (72.4%) | **0.010** |
| Not married/Living alone | 362 | 288 (79.6%) |  |
| Nationality |  |  | 0.464 |
| Foreign | 87 | 68 (78.2%) |  |
| Italian | 1598 | 1183 (74.0%) |  |
| Permanent address |  |  | 0.478 |
| In a region capital | 904 | 661 (73.1%) |  |
| In a province capital | 63 | 46 (73.0%) |  |
| Other | 705 | 534 (75.7%) |  |
| Education |  |  | 0.541 |
| No education | 30 | 22 (73.3%) |  |
| Primary | 207 | 150 (72.5%) |  |
| Secondary | 828 | 633 (76.4%) |  |
| Higher | 226 | 165 (73.0%) |  |

**Supplemental Table 8.** MRA prescription for patients without contraindications in the overall cohort.

| Variables | Total patients (n = 1730) | Patients with MRA | p-value |
| --- | --- | --- | --- |
| Demographics/organizational/socioeconomic |  |  |  |
| Age |  |  | 0.818 |
| > 75 years | 672 | 198 (29.5%) |  |
| ≤ 75 years | 1058 | 305 (28.8%) |  |
| Sex |  |  | 0.223 |
| Male | 1315 | 372 (28.3%) |  |
| Female | 415 | 131 (31.6%) |  |
| Civil staus |  |  |  |
| Married/Living with someone | 936 | 266 (28.4%) | 0.498 |
| Not married/Living alone | 379 | 100 (26.4%) |  |
| Nationality |  |  | 0.914 |
| Foreign | 93 | 28 (30.1%) |  |
| Italian | 1637 | 475 (29.0%) |  |
| Permanent address |  |  | 0.393 |
| In a region capital | 932 | 266 (28.5%) |  |
| In a province capital | 65 | 15 (23.1%) |  |
| Other | 719 | 218 (30.3%) |  |
| Education |  |  | 0.116 |
| No education | 30 | 9 (30.0%) |  |
| Primary | 208 | 71 (34.1%) |  |
| Secondary | 857 | 223 (26.0%) |  |
| Higher | 231 | 60 (26.0%) |  |

**Supplemental Table 9.** SGLT2i prescription for patients without contraindications after June 2022.

| Variables | Total patients (n = 582) | Patients with SGLT2i | p-value |
| --- | --- | --- | --- |
| Demographics/organizational/social |  |  |  |
| Age |  |  | **0.001** |
| > 75 years | 236 | 90 (38.1%) |  |
| ≤ 75 years | 346 | 183 (52.9%) |  |
| Sex |  |  | 0.158 |
| Male | 444 | 216 (48.6%) |  |
| Female | 138 | 57 (41.3%) |  |
| Civil staus |  |  |  |
| Married/Living with someone | 266 | 134 (50.4%) | 0.914 |
| Not married/Living alone | 128 | 63 (49.2%) |  |
| Nationality |  |  | 1.000 |
| Foreign | 36 | 17 (47.2%) |  |
| Italian | 546 | 256 (46.9%) |  |
| Permanent address |  |  | 0.393 |
| In a region capital | 321 | 143 (44.5%) |  |
| In a province capital | 19 | 10 (52.6%) |  |
| Other | 236 | 118 (50.0%) |  |
| Education |  |  | **0.026** |
| No education | 11 | 4 (36.4%) |  |
| Primary | 63 | 22 (34.9%) |  |
| Secondary | 267 | 144 (53.9%) |  |
| Higher | 77 | 39 (50.6%) |  |

**Supplemental Table 10.** Diuretics prescription for patients without contraindications in the overall cohort.

| Variables | Total patients (n = 1730) | Patients with  diuretics | p-value |
| --- | --- | --- | --- |
| Demographics/organizational/socioeconomic |  |  |  |
| Age |  |  | **0.001** |
| > 75 years | 672 | 614 (91.4%) |  |
| ≤ 75 years | 1058 | 910 (86.0%) |  |
| Sex |  |  | 0.121 |
| Male | 1315 | 1149 (87.4%) |  |
| Female | 415 | 375 (90.4%) |  |
| Civil staus |  |  |  |
| Married/Living with someone | 936 | 813 (86.9%) | 0.353 |
| Not married/Living alone | 379 | 337 (88.9%) |  |
| Nationality |  |  | 0.604 |
| Foreign | 93 | 84 (90.3%) |  |
| Italian | 672 | 1440 (88.0%) |  |
| Permanent address |  |  | 0.959 |
| In a region capital | 932 | 819 (87.9%) |  |
| In a province capital | 65 | 57 (87.7%) |  |
| Other | 719 | 635 (88.3%) |  |
| Education |  |  | **0.005** |
| No education | 30 | 24 (80.0%) |  |
| Primary | 208 | 197 (94.7%) |  |
| Secondary | 857 | 738 (86.1%) |  |
| Higher | 231 | 201 (87.0%) |  |

**Supplemental Figure 1.** Distributions of the mOMT score across gender, civil status, permanent address, birthplace, and education level.


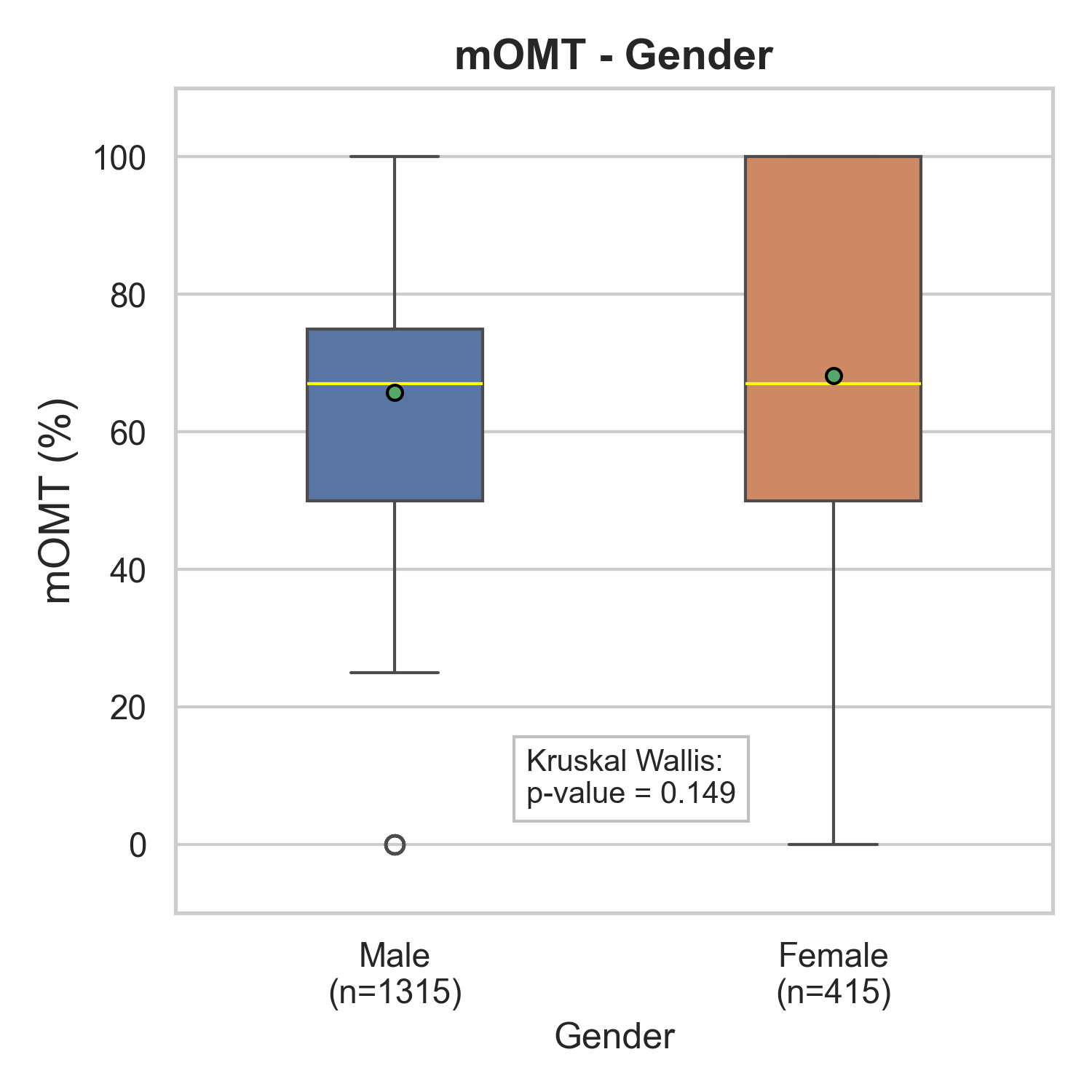

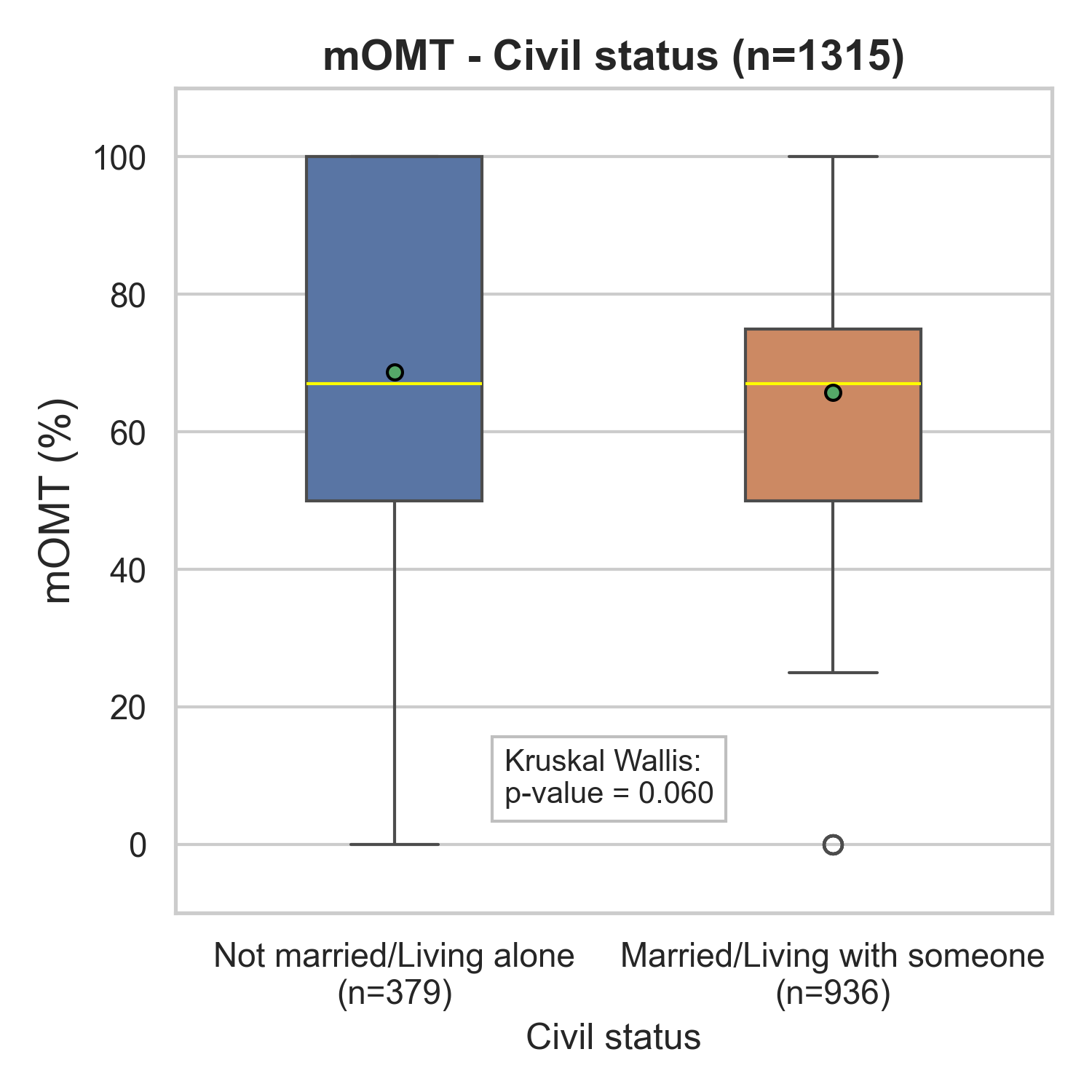


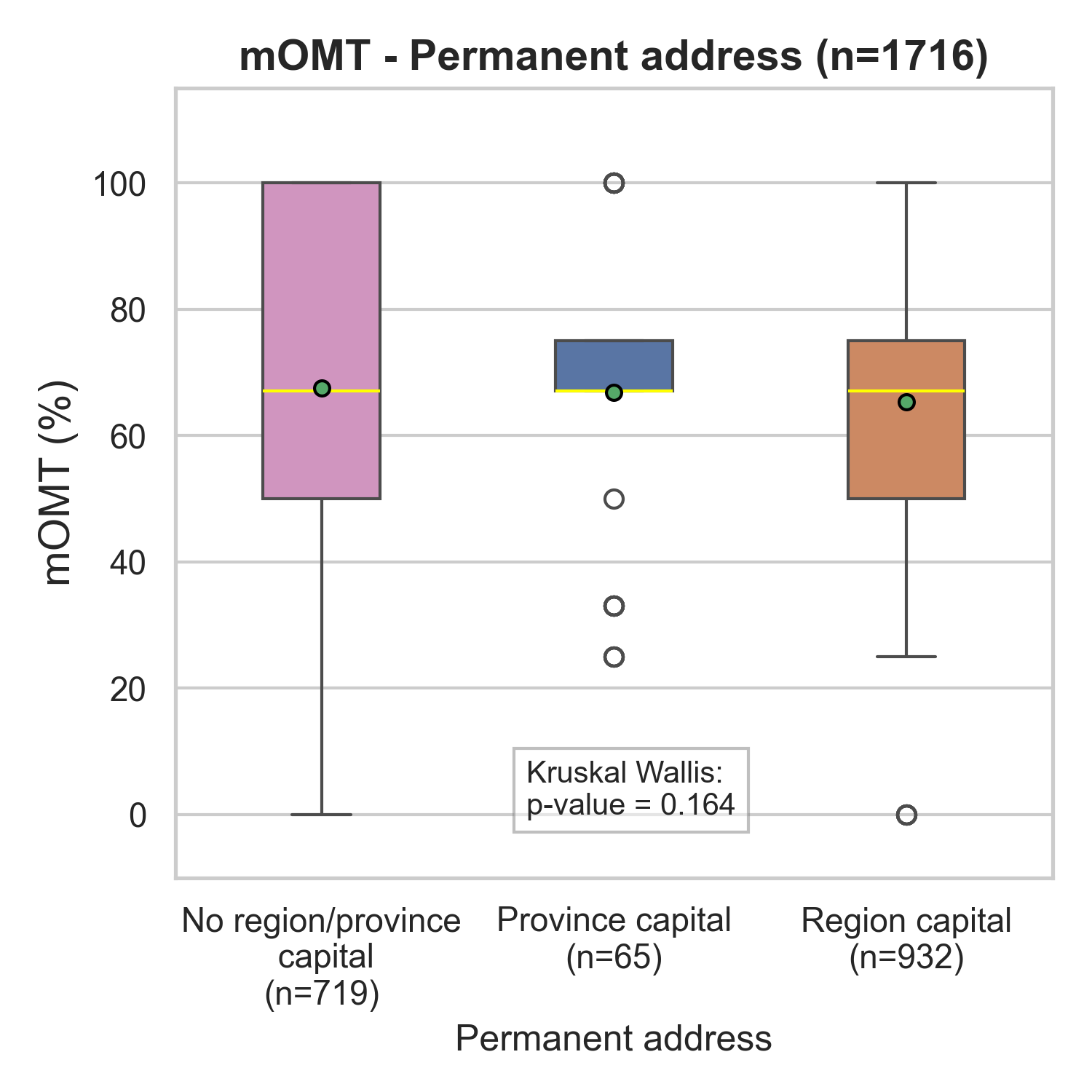

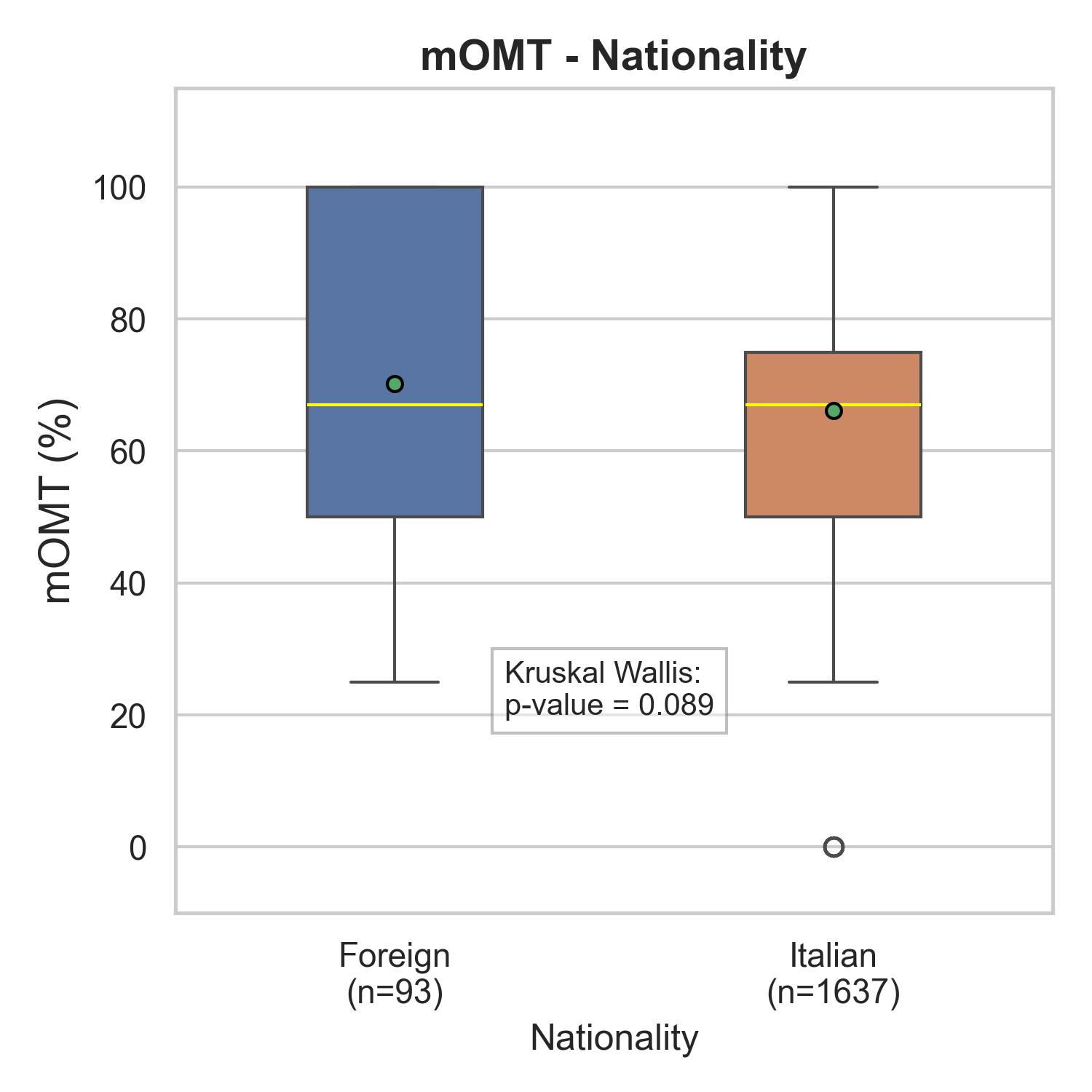


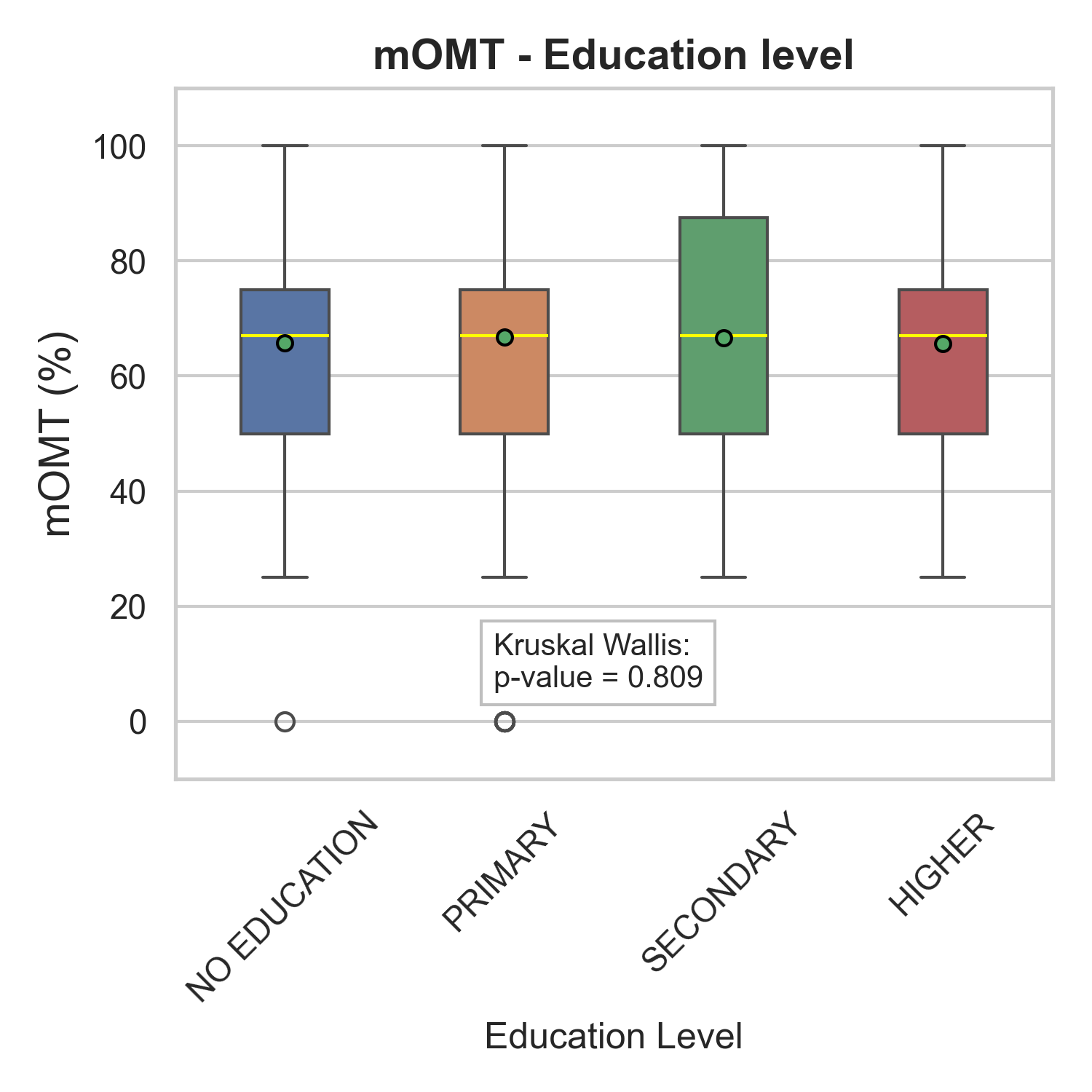


In boxplots, the yellow line represents the mean value while the green circle the median value.

*Abbreviation: mOMT: modified optimal medical therapy.*

**Supplemental Figure 2.** Calibration plots for mOMT score and ACEi/ARB/ARNI prescription.


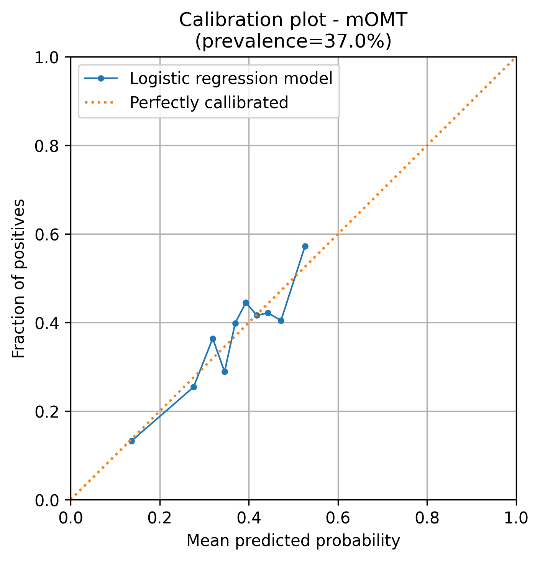


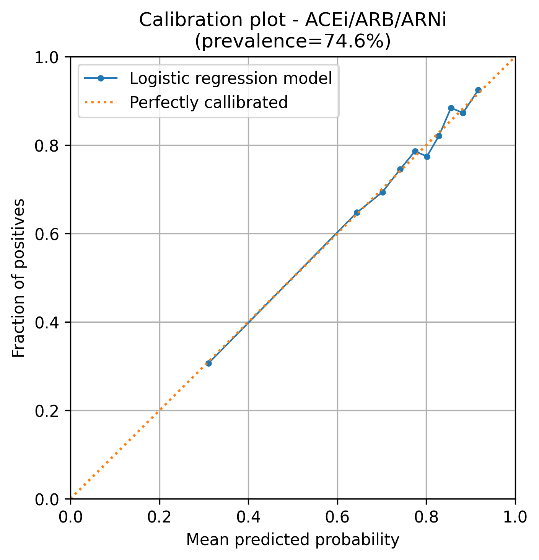


*Abbreviations: ACEi/ARB/ARNI, Angiotensin-Converting Enzyme inhibitors, Angiotensin II Receptor Blockers, Angiotensin Receptor-Neprilysin Inhibitors; mOMT, modified optimal medical therapy*
